# A Comparative Analysis of Constitutive Promoters Located in Adeno-Associated Viral Vectors

**DOI:** 10.1371/journal.pone.0106472

**Published:** 2014-08-29

**Authors:** Lkhagvasuren Damdindorj, Sivasundaram Karnan, Akinobu Ota, Ekhtear Hossain, Yuko Konishi, Yoshitaka Hosokawa, Hiroyuki Konishi

**Affiliations:** Department of Biochemistry, Aichi Medical University School of Medicine Nagakute, Aichi, Japan; Rush University Medical Center, United States of America

## Abstract

The properties of constitutive promoters within adeno-associated viral (AAV) vectors have not yet been fully characterized. In this study, AAV vectors, in which enhanced GFP expression was directed by one of the six constitutive promoters (human β-actin, human elongation factor-1α, chicken β-actin combined with cytomegalovirus early enhancer, cytomegalovirus (CMV), simian virus 40, and herpes simplex virus thymidine kinase), were constructed and introduced into the HCT116, DLD-1, HT-1080, and MCF-10A cell lines. Quantification of GFP signals in infected cells demonstrated that the CMV promoter produced the highest GFP expression in the six promoters and maintained relatively high GFP expression for up to eight weeks after infection of HCT116, DLD-1, and HT-1080. Exogenous human *CDKN2A* gene expression was also introduced into DLD-1 and MCF-10A in a similar pattern by using AAV vectors bearing the human β-actin and the CMV promoters. The six constitutive promoters were subsequently placed upstream of the neomycin resistance gene within AAV vectors, and HCT116, DLD-1, and HT-1080 were infected with the resulting vectors. Of the six promoters, the CMV promoter produced the largest number of G418-resistant colonies in all three cell lines. Because AAV vectors have been frequently used as a platform to construct targeting vectors that permit gene editing in human cell lines, we lastly infected the three cell lines with AAV-based targeting vectors against the human *PIGA* gene in which one of the six promoters regulate the neomycin resistance gene. This assay revealed that the CMV promoter led to the lowest *PIGA* gene targeting efficiency in the investigated promoters. These results provide a clue to the identification of constitutive promoters suitable to express exogenous genes with AAV vectors, as well as those helpful to conduct efficient gene targeting using AAV-based targeting vectors in human cell lines.

## Introduction

A variety of cellular and viral constitutive promoters have been used in expression vectors to introduce exogenous genes into cells. These promoters have distinct advantages; for example, cytomegalovirus (CMV) early enhancer/promoter directs a high level of transient gene expression in many types of cells [1]–[4]. However, a potential drawback of the CMV promoter is that it is prone to silencing over time after being transduced into the genome of host cells [4]–[9], although the activity of this promoter may vary depending on host cells and experimental settings [10], [11]. Some other constitutive promoters, including human β-actin (*hACTB*), human elongation factor-1α (*hEF-1α*), and cytomegalovirus early enhancer/chicken β-actin (CAG) promoters, have shown their merits in sustaining stable gene expression for long periods of time [4]–[8], [12]–[17]. However, most of the studies characterizing constitutive promoters within expression vectors have been performed using plasmid, adenoviral, retroviral, or lentiviral vectors as a platform for experiments.

Adeno-associated viral (AAV) vector is one of the promising vectors for gene transfer strategies in clinical use [18], [19]. Native AAV, from which this type of vector is derived, is likely nonpathogenic in human. In addition, commonly used AAV vectors produce no viral proteins because all native endogenous AAV genes are removed. The majority of AAV vectors introduced into cells are likely to be retained as extrachromosomal DNA and not integrated into the genome of host cells [20]. Thus, AAV vectors are considered to be relatively safe viral vectors that infrequently modify host genomes. In regard to promoters used in AAV vectors, a previous study demonstrated that the *EF-1α* promoter directs more stable long-term gene expression than the CMV promoter within AAV vectors in the HT-29 colon cancer cell line [21]. However, to our knowledge, there have been few studies that systematically compare long-term transcriptional activities of more than two constitutive promoters in AAV vectors. In this study, therefore, we addressed the transient and long-term transcriptional activities of six constitutive promoters located in AAV vectors in human cell lines.

As well as vehicles for gene delivery, AAV vectors have also been used as backbone for gene targeting vectors that allow for efficient gene editing in human cell lines [22]. In AAV-mediated gene targeting, cells are infected with AAV-based targeting vectors bearing an antibiotic resistance gene and then subjected to appropriate antibiotic selection. Surviving colonies are recovered and screened for gene-targeted cell clones. Because of low homologous recombination efficiency in most human cell lines, it is usually necessary to screen a large number of colonies to successfully identify gene-targeted cell clones, even though the use of AAV vectors permits relatively efficient gene targeting. A system that enables simple production of a large number of antibiotic-resistant colonies could reduce the intense labor involved in gene targeting in human cell lines. Strong and sustained expression of an antibiotic resistance gene from the targeting vector should, in theory, enhance colony formation under antibiotic selection. In this study, we thus evaluated the formation of antibiotic-resistant colonies achieved by a series of AAV-based targeting vectors in which one of the six constitutive promoters directs the antibiotic resistance gene. This experiment also allowed us to address whether the choice of a particular promoter driving an antibiotic resistance gene in AAV-based targeting vectors has an impact on gene targeting efficiency in human cell lines.

## Materials and Methods

### Vector Construction

For GFP assays, a series of AAV plasmids harboring the enhanced GFP (EGFP) gene driven by one of the six constitutive promoters was constructed. To create AAV plasmids carrying the *hACTB*, *hEF-1α*, CAG, CMV, herpes simplex virus thymidine kinase (HSV-TK) promoters, or the *hACTB* promoter in the reverse direction (*hACTB*-R), plasmids expressing EGFP under the control of respective promoters [4] were digested with a restriction endonuclease *Ssp*I to remove a fragment including an f1 origin and a bacterial promoter upstream of the kanamycin resistance/neomycin resistance (Kan^R^/Neo^R^) gene (Fig. S1). The remaining DNA fragments containing both the Neo^R^ and the EGFP gene cassettes were ligated to the backbone of pAAV-MCS (Agilent Technologies) at the NotI–NotI site. An AAV plasmid carrying the EGFP gene without a promoter (no promoter; N.P.) was constructed in the same manner using a control plasmid created in our previous study [4]. To create an AAV plasmid in which the simian virus 40 early gene (SV40) promoter drives EGFP, the SV40 promoter was isolated from the 5A plasmid (a platform vector to construct targeting vectors expressing Neo^R^ under the control of the SV40 promoter [23]; a gift from Dr. Ben H. Park) and then inserted upstream of the EGFP gene within the AAV plasmid.

To construct AAV vectors in which either the CMV or the *hACTB* promoter directs the *CDKN2A* gene with H83Y (247C>T) substitution, the above-described AAV vectors expressing EGFP from the CMV and the *hACTB* promoters were digested with AgeI and BamHI to remove the EGFP gene. The remaining portion of the vectors were ligated with the coding sequence of *CDKN2A* (H83Y) amplified as an AgeI-BamHI fragment by PCR. DNA fragments within the plasmids generated by PCR amplification was verified by DNA sequencing.

For *PIGA* gene targeting assays, a series of AAV-based targeting vectors in which Neo^R^ is driven by one of the six constitutive promoters was created. A *PIGA*-targeting vector bearing the CMV promoter was constructed as previously described [24]. To generate a *PIGA*-targeting vector bearing the *hACTB* promoter, promoter-trap *PIGA*-targeting vector [24] was digested with *Bam*HI and *Hin*dIII. A fragment consisting of an AAV backbone and both homology arms was recovered and ligated with a PCR-amplified *Bam*HI–*Hin*dIII fragment containing the *hACTB* promoter. The resultant plasmid was cleaved with *Hin*dIII and *Cla*I and then ligated with a *Hin*dIII–*Cla*I fragment containing the Neo^R^ gene and a synthetic polyadenylation (polyA) site derived from pCI-neo (Promega).

For the other versions of AAV-based *PIGA*-targeting vectors, the *PIGA*-targeting vector bearing the *hACTB* promoter was digested with *Bam*HI and *Hin*dIII to remove the *hACTB* promoter. The remaining portion of the vector was ligated with respective promoter fragments isolated from the above-described AAV plasmids in which promoters drove EGFP. To create a control vector without a promoter, the *PIGA*-targeting vector bearing the *hACTB* promoter was digested with *Bam*HI and *Hin*dIII, blunt-ended, and self-ligated. For targeting vectors used in the hygromycin resistance gene (Hyg^R^)–EGFP fusion gene-based system, the SV40 promoter within SV40p TV [25] was replaced with respective constitutive promoters tested in this study.

### Cell culture

All cell lines were obtained from ATCC, except for the HT-1080 cell line that was obtained from Health Science Research Resources Bank, Japan. Cell lines other than MCF-10A were cultured in Dulbecco’s modified Eagle’s medium (Wako) supplemented with 5% fetal bovine serum (Biowest) and 1% penicillin/streptomycin (Wako). MCF-10A cells were cultured in a condition described previously [23]. AAV particles (serotype 2) were produced by cotransfecting 293T cells with one of the aforementioned AAV plasmids, along with the pAAV-RC and pHelper plasmids included in the AAV Helper-Free System (Agilent Technologies) as described previously [26]. TransIT-LT1 (Mirus Bio) was used for transfection as per manufacturer’s instructions. Infection of cells with AAV vectors was carried out as previously described [26] at a multiplicity of infection (MOI) of 1×10^4^ unless otherwise noted. Copy numbers of AAV vectors were determined according to a procedure based on quantitative real-time PCR [24]. Antibiotic selection with G418 (Life Technologies) was performed at concentrations of 0.4 mg/ml (HCT116), 1 mg/ml (DLD-1), 0.4 mg/ml (HT-1080), and 0.12 mg/ml (MCF-10A) unless otherwise noted. Fluorescence flow cytometric (FCM) analyses of the cells were performed using a FACSCanto II flow cytometer and FACSDiva software version 5.0.2 (BD Biosciences).

### FCM-based GFP assay

FCM-based GFP assays were performed as described previously [4] with some modifications. In brief, cell lines were seeded in 10-cm dishes at approximately 30% confluence and infected with AAV vectors expressing EGFP on the following day. Two days after infection, cells were treated with trypsin, and half of the cells were processed for FCM analysis. A population of uninfected cells within each infectant was determined on the basis of an FL1-A (530±15 nm) versus FL2-A (585±21 nm) dot plot and excluded from the analysis. The other half of the cells were propagated in culture medium containing G418 in a new 10-cm dish and subjected to a time-course GFP assay in which GFP signals were measured over time in the same fashion.

### Quantitative reverse transcriptase (qRT)-PCR

Cell lines were seeded in 6-well plates at a density of 1×10^4^ cells/well and infected with AAV vectors expressing *CDKN2A* (H83Y) on the next day. Two days after infection, total RNA was extracted from the cells in each well and processed for the synthesis of complementary DNA (cDNA) with the aid of High Capacity cDNA Reverse Transcription Kit (Life Technologies) as per the manufacturer’s instructions. qRT-PCR using the cDNA as templates was performed in triplicate with StepOnePlus Real-Time PCR System (Life Technologies) and SYBR Green Dye (Takara Bio). Oligonucleotide primers used for qRT-PCR are listed in Table S1. A standard curve was generated for each session using serially diluted samples, and gene expression in each sample was determined in reference to the standard curve. *CDKN2A* gene expression in each sample was expressed after normalization to *GAPDH*.

### Colony formation assay

Cell lines seeded in 25-cm^2^ flasks (1×10^4^ cells/flask) were infected with AAV-based targeting vectors and incubated in culture medium containing G418 for two weeks. Surviving colonies were fixed/stained with 3.7% formaldehyde (Sigma-Aldrich) containing 0.2% (wt/vol) crystal violet (Sigma-Aldrich) and processed for counting. HCT116 cells were selected with G418 at a concentration of 0.8 mg/ml.

### *PIGA* gene targeting assay

*PIGA* gene targeting assay was performed as previously described [24] with slight modifications. In brief, cell lines seeded in 75-cm^2^ flasks (1×10^6^ cells/flask) were infected with AAV-based *PIGA*-targeting vectors as described previously [26]. After 2–3 weeks of G418 selection, the bulk population of cells was dissociated, stained with FLAER (Pinewood Scientific Services) according to the manufacturer’s instructions, and subjected to fluorescence FCM analyses. An AAV vector bearing the Neo^R^ gene cassette and a 1 kb fragment consisting of the internal portion of SV40 large T was used as a control vector (V.C.) in this assay. Cell lines were infected with V.C., selected with G418, and then stained with FLAER to obtain FLAER-positive controls in FCM analyses. For FLAER-negative controls, unstained V.C. infectants were used.

### Hyg^R^–EGFP-based gene targeting assay

Prior to the gene targeting assay based on the Hyg^R^–EGFP reporter system, a DLD-1-derived reporter clone established in the previous study [25] was subjected to single cell cloning by limiting dilution, and a newly isolated clone was used in this study. The quantification of gene targeting efficiency with this system was performed as previously described [25]. Briefly, the isolated reporter clone was plated in 75-cm^2^ flasks (5×10^5^ cells/flask) and infected with AAV-based targeting vectors. The infected cells were then selected with G418 for 1–2 weeks and harvested to determine GFP positive ratios using fluorescence FCM performed with a FACSCanto II flow cytometer.

### Statistical analyses

All statistical analyses were performed with Intercooled Stata (Stata). One-way analysis of variance (ANOVA) followed by Scheffe’s post-hoc test was used to analyze transient GFP signals, qRT-PCR data, and the numbers of G418-resistant colonies in each cell line. Data for each cell line acquired in GFP time-course studies were initially analyzed with two-way ANOVA with two independent variables (IVs), “promoter” and “week”. As a result, the main effects of “promoter” were significant and there was no interaction between two IVs in the HCT116, DLD-1, and MCF-10A cell lines. Thus, the data of these cell lines were next analyzed with Scheffe’s post-hoc test to evaluate the difference between individual promoters. In the HT-1080 cell line, the main effect of “promoter” was significant and there was an interaction between two IVs in the initial analysis with two-way ANOVA. Thus, the data of this cell line at respective weeks were next analyzed separately using one-way ANOVA with Scheffe’s post-hoc test.

Correlations between “gene targeting efficiencies” and “the numbers of G418-resistant colonies”, as well as between “gene targeting efficiencies” and “long-term GFP signal intensities”, were indicated by Pearson’s correlation coefficients. GFP signal intensities quantified four weeks after infection with respective AAV vectors were used as “long-term GFP signal intensities”. In all statistical analyses, a *p*-value of less than 0.05 was considered to be significant.

## Results

### Transient transcriptional activities of six constitutive promoters located in AAV vectors

**Figure 1:**
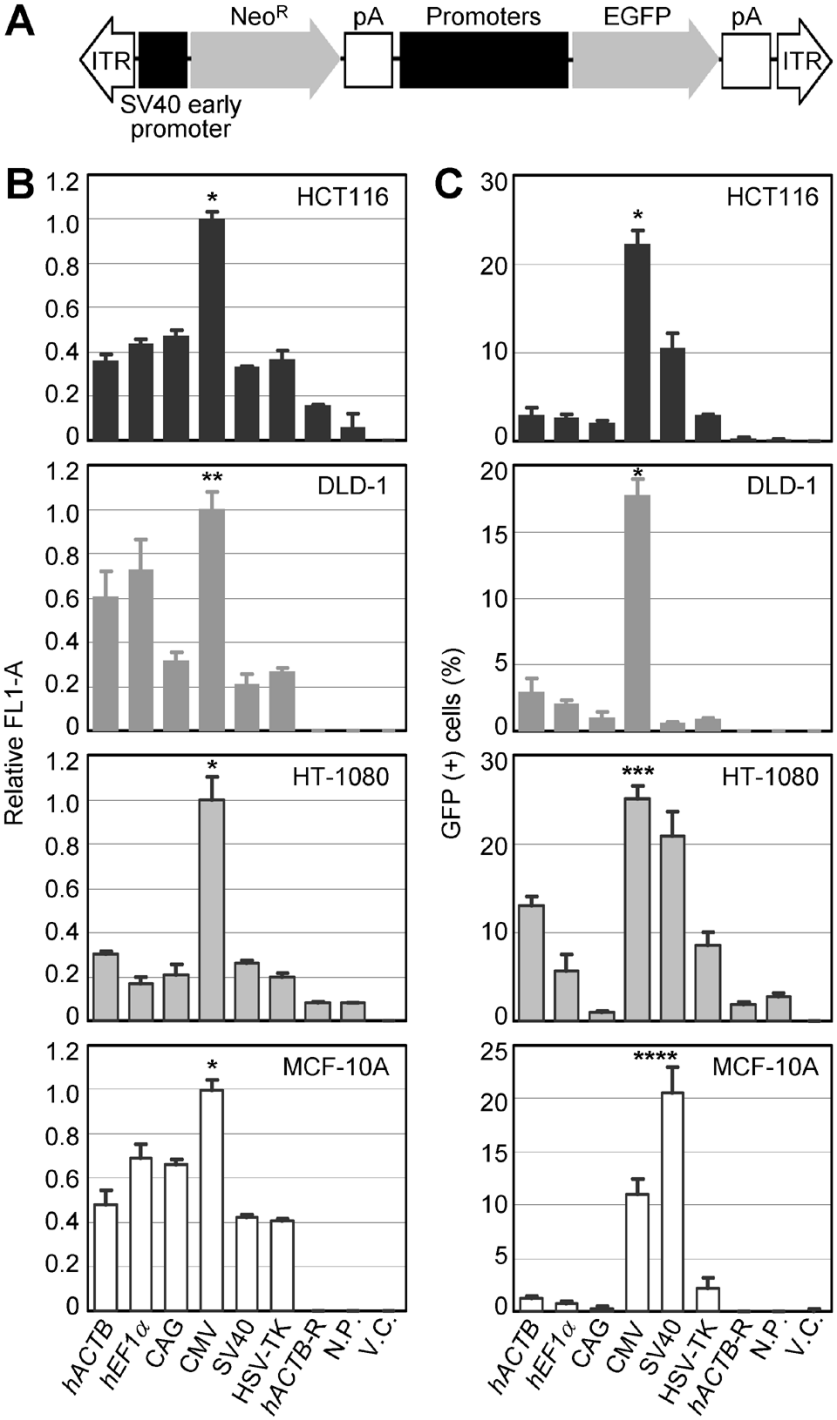
Transient GFP expression driven by various constitutive promoters in AAV vectors. (**A**) Schematic representation of AAV vectors used for GFP assays. “Promoters” indicates the position where one of the six promoters was incorporated. ITR, inverted terminal repeat; SV40, simian virus 40; Neo^R^, neomycin phosphotransferase gene; pA, polyadenylation site; EGFP, enhanced GFP. The diagram is not drawn to scale. (**B, C**) Transient GFP expression in human cell lines. Cell lines denoted within bar graphs were infected with the AAV vectors shown in (A), and GFP signals were quantified by fluorescence FCM after 2-days incubation. Shown are mean GFP intensity (acquired as mean FL1-A) for each infectant relative to data obtained with the CMV promoter (B) and percentage of GFP positive cells within each infectant (C) (mean ± s.e.m.; n = 3). *hACTB*, human β-actin; *hEF-1α*, human elongation factor-1α; CAG, cytomegalovirus early enhancer/chicken β-actin; CMV, cytomegalovirus; HSV-TK, herpes simplex virus thymidine kinase; *hACTB*-R, *hACTB* reverse direction; N.P., no promoter; V.C., vector control. For V.C., an unrelated AAV vector harboring no EGFP gene was used. *Significantly higher than any of the other promoters and controls (*p*<0.001 for all pairs). **Significantly higher than CAG, SV40, HSV-TK, *hACTB*-R, N.P. and V.C. (*p* = 0.001 versus CAG; *p*<0.001 versus the others). ***Significantly higher than *hACTB*, *hEF-1α*, CAG, HSV-TK, *hACTB*-R, N.P. and V.C. (*p* = 0.004 versus *hACTB*; *p*<0.001 versus the others). ****The data for SV40 is the highest (*p* = 0.002 versus CMV; *p*<0.001 versus the others), and those for CMV is the second highest (*p* = 0.001 versus *hACTB* and *hEF-1α*; *p* = 0.003 versus HSV-TK; *p*<0.001 versus CAG, *hACTB*-R, N.P. and V.C) among the investigated promoters and controls. *p*-values were determined based on Scheffe’s post hoc test.

We initially constructed a series of AAV vectors in which the EGFP gene was expressed under the control of one of the six constitutive promoters. The investigated promoters includes those commonly used to express exogenous genes in mammalian cells (the *hACTB* [4], [14], [27], [28], *hEF-1α* [5], [7], [15], [21], CAG [2], [17], [29], CMV [1], [30], [31], and SV40 [30], [32]–[34] promoters) as well as the HSV-TK [34]–[36] promoter as a representative of relatively weak constitutive promoters (Fig. 1A). These AAV vectors were identical except for the promoters driving EGFP gene expression. For negative controls, we constructed two additional AAV vectors carrying either *hACTB*-R or N.P. upstream of the EGFP gene. In a previous study, we constructed two plasmid vectors carrying the same genetic modules (the EGFP gene with either *hACTB*-R or N.P.) and found that these plasmids yield no appreciable GFP signals upon transfection into human cell lines [4].

To address transient transcriptional activities of the six constitutive promoters, we infected four human cell lines derived from three distinct tissue types with the constructed AAV vectors and quantified GFP expression levels by fluorescence FCM analyses after 2-days incubation. The infected cell lines include the HCT116 and the DLD-1 colon cancer cell lines, the HT-1080 fibrosarcoma cell line, and a non-cancerous MCF-10A breast epithelial cell line. As a result, the GFP signal elicited by the CMV promoter was significantly greater compared with those elicited by any of the other five promoters in HCT116, HT-1080, and MCF-10A (Figs. 1B and S2). In the DLD-1 cell line, the CMV promoter elicited GFP signal significantly more than three non-cellular promoters (CAG, SV40, and HSV-TK) but not more than two human cellular promoters (*hACTB* and *hEF-1α*).

As for the ratio of GFP positive cells, the CMV promoter yielded significantly larger population of GFP positive cells than any of the other five constitutive promoters in HCT116 and DLD-1, and than any of the five constitutive promoters except for the SV40 promoter in HT-1080 (Fig. 1C). In the MCF-10A cell line, the SV40 and the CMV promoters yielded the largest and the second largest fractions of GFP positive cells, respectively, among the tested promoters. These results indicated that the levels of transcription elicited by respective promoters located in AAV vectors vary depending on recipient cell lines. However, the CMV promoter within an AAV vector conferred relatively strong transcriptional activity in multiple human cell lines at two days after infection.

### Long-term transcriptional activities of six constitutive promoters located in AAV vectors

**Figure 2:**
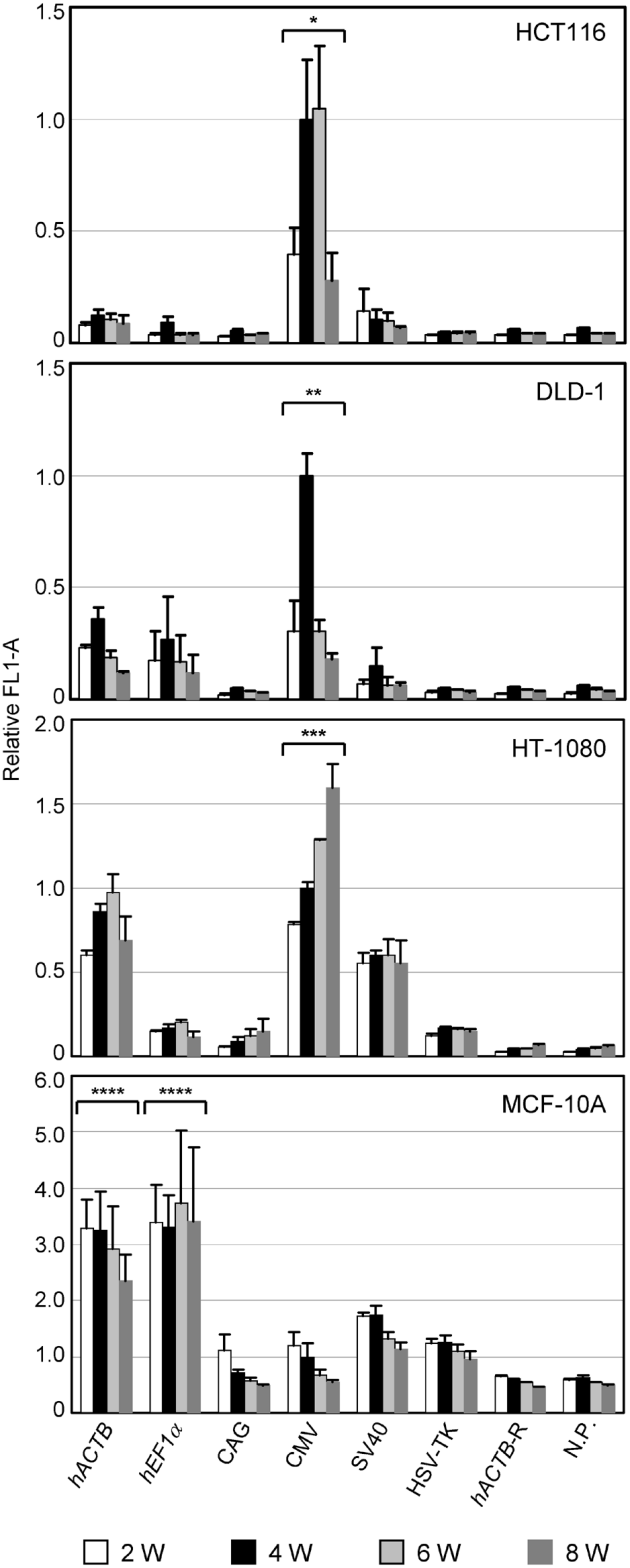
Time-course study of GFP signals expressed by various constitutive promoters in AAV vectors. Cell lines indicated within bar graphs were infected with the AAV vectors depicted in Figure 1A and processed for fluorescence FCM after 2-, 4-, 6-, and 8-weeks culture in the presence of G418. Mean GFP intensity (acquired as mean FL1-A) for each infectant is indicated relative to data obtained with the CMV promoter four weeks after infection (mean ± s.e.m.; n = 3). For abbreviations, refer to the legend for Figure 1. *Significantly higher than any of the other promoters and controls (*p*<0.001 for all pairs). **Significantly higher than any of the other promoters and controls (*p* = 0.004, versus *hACTB*; *p* = 0.002, versus *hEF-1α*; *p*<0.001 versus the others). ***Significantly higher than *hACTB* in two weeks (*p* = 0.01) and eight weeks (*p*<0.001), and than the other promoters and controls in any of four time points (*p*<0.001, versus all the others in any of four time points, except for the comparison of CMV versus SV40 in two weeks, *p* = 0.001). ****Significantly higher than CAG, CMV, SV40, HSV-TK, *hACTB*-R, and N.P. (*p* = 0.001 for *hACTB* versus SV40; *p*<0.001 for all the other pairs). *p*-values were determined based on Scheffe’s post hoc test.

To address long-term transcriptional activities of the six constitutive promoters, we propagated the cell lines infected with AAV vectors in the presence of G418 and measured GFP signals over time for up to eight weeks by fluorescence FCM analyses. This assay demonstrated that the CMV promoter produces the highest GFP signals among the investigated promoters for long time periods in the HCT116, DLD-1, and HT-1080 cell lines (Fig. 2). However, in the MCF-10A cell line, the use of the CMV promoter resulted in a level of GFP expression among the weakest in the six constitutive promoters. Two human cellular promoters (*hACTB* and *hEF-1α*) directed significantly higher GFP signals than the other promoters in the MCF-10A cell line. In each cell line, some promoters elicited a level of GFP signals not significantly greater than negative controls, suggesting their minimal long-term transcriptional activities comparable with background levels. However, the long-term transcriptional activities of the constitutive promoters varied depending on recipient cell lines, similar to their short-term activities. Overall, these assays demonstrated that the CMV promoter in the AAV vector directed the strongest long-term GFP signal in three out of the four cell lines analyzed in this study.

### GFP signals emitted from the cells infected with an EGFP-expressing AAV vector at various MOIs

**Figure 3:**
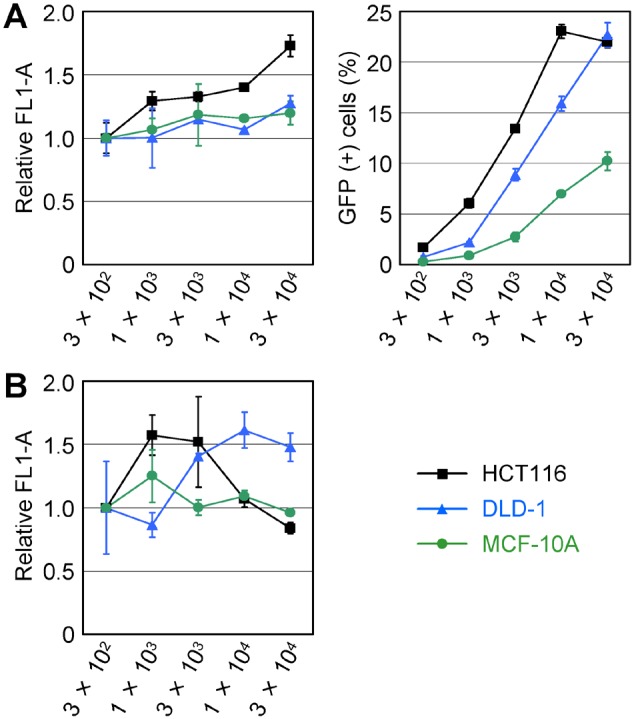
The impact of the MOI of an AAV vector on GFP expression level. The HCT116, DLD-1, and MCF-10A cell lines were infected with various MOIs of an AAV vector expressing EGFP under the control of the CMV promoter, and GFP expression was quantified by fluorescence FCM (mean ± s.e.m.; n = 3). X-axes indicate MOIs of the AAV vector. (**A**) Fluorescence FCM analyses performed after two days of culture post-infection without G418. (left) Mean GFP intensities (acquired as mean FL1-A) for respective infectants are shown relative to the data obtained with the cells infected at an MOI of 3×10^2^. (right) Percentage of GFP positive cells in each infectant. (**B**) Fluorescence FCM analyses performed after four weeks of culture post-infection in the presence of G418. Data are shown in the same manner with the left panel of (A).

We next infected the HCT116, DLD-1, and MCF-10A cell lines with various amounts of an AAV vector in which the CMV promoter regulated the EGFP gene, and detected GFP signals expressed from the infectants using fluorescence FCM at two days and four weeks after infection. In the GFP assay at two days after infection, we detected marginally stronger GFP signals expressed from the cells infected at higher MOIs in all three cell lines (Figs. 3A and S3). However, infection at higher MOIs led to obviously larger populations of GFP positive cells, probably reflecting the infection efficiency achieved by each amount of AAV vector. The infected cells were then propagated for four weeks in the presence of G418, and again analyzed by fluorescence FCM to address GFP signal intensities. After four weeks of propagation, we found no correlation between the amount of AAV vector used for infection and the intensity of GFP signals emitted from the cells (Fig. 3B).

### Expression of the *CDKN2A* gene regulated by the CMV and the *hACTB* promoters within an AAV vector

We next sought to confirm the transcriptional activities of constitutive promoters located in AAV vectors using a different experimental setting. To this end, human *CDKN2A* (*p16*/*INK4A*) gene under the control of the CMV or the *hACTB* promoter was introduced into cells using an AAV vector, and the expression of the *CDKN2A* gene was measured by qRT-PCR after 2-days incubation without G418 as well as 4-weeks incubation with G418. The human *CDKN2A* gene was chosen in this analysis as an example of human endogenous genes potentially relevant for clinical use against cancer cells [37]–[40]. The DLD-1 and the MCF-10A cell lines were used for recipients of the AAV vectors, because the expression of endogenous *CDKN2A* gene in these cell lines was abrogated by the methylation of promoter region [41] and the homozygous deletion of the gene, respectively, allowing for an accurate measurement of the exogenous *CDKN2A* gene expression. To alleviate the impact of *CDKN2A* overexpression on cell cycle progression [42], a H83Y variant form of the *CDKN2A* gene was utilized for this analysis.

**Figure 4:**
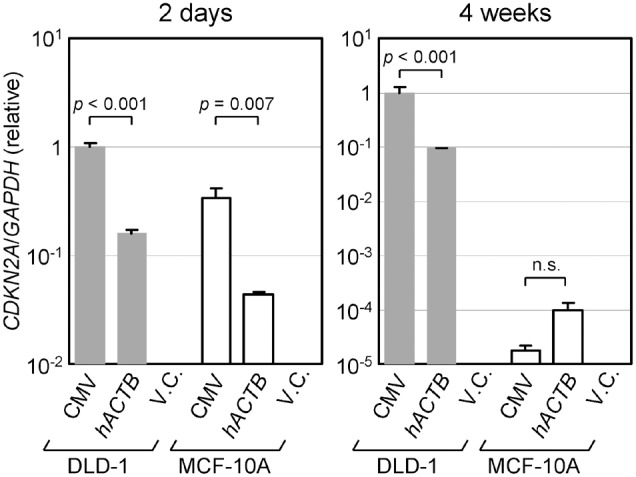
qRT-PCR analyses of *CDKN2A* gene expression. The indicated cell lines were infected with an AAV vector carrying a *CDKN2A* gene (H83Y) downstream of the CMV or the *hACTB* promoter. The cells were then cultured for two days without G418 (left) or for four weeks with G418 (right), and total RNA was extracted from each infectant, converted to cDNA, and used as a template in qRT-PCR. The expression level of *CDKN2A* was normalized to that of the *GAPDH* gene in each sample and shown relative to the data of the DLD-1 cell line obtained with the CMV promoter (mean ± s.e.m.; n = 3). An AAV vector carrying no *CDKN2A* gene was used as V.C. *p*-values were determined based on Scheffe’s post hoc test and denoted in the graph.

Two days after infection, the CMV promoter directed significantly higher *CDKN2A* expression compared to the *hACTB* promoter both in the DLD-1 and the MCF-10A cell lines (Fig. 4). In the DLD-1 cell line, the *CDKN2A* expression induced by the CMV promoter at four weeks after infection was still significantly higher than that induced by the *hACTB* promoter. However, in the MCF-10A cell line, the difference in the level of induced *CDKN2A* expression found at two days after infection was diminished, and the *CDKN2A* expression induced by the *hACTB* promoter was marginally higher, at four weeks after infection. These results were largely consistent with our previous data obtained with EGFP-expressing AAV vectors shown in Figures 1 and 2.

### G418-resistant colony formation induced by a series of AAV vectors in which different promoters regulate Neo^R^

**Figure 5:**
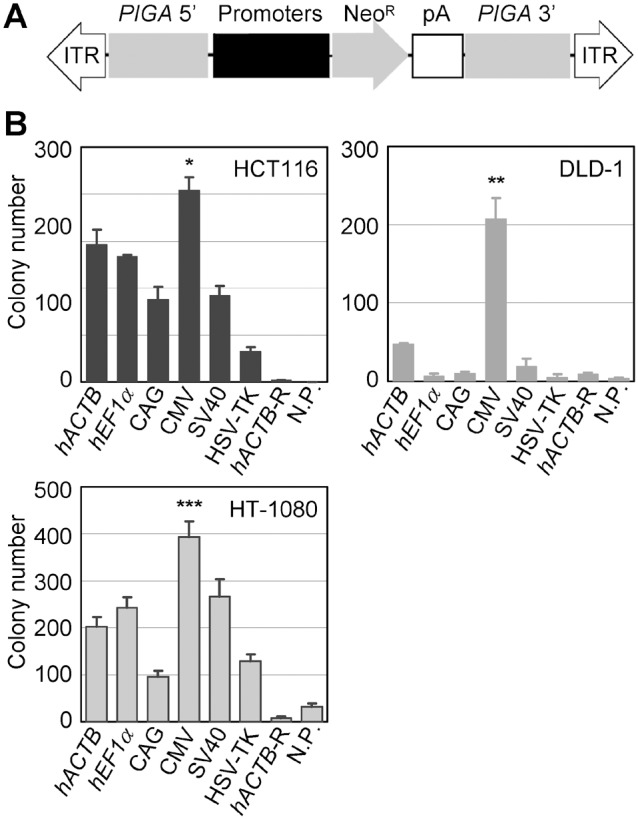
Formation of G418-resistant colonies by the infection of AAV vectors in which the Neo^R^ gene is directed by various promoters. (**A**) Schematic representation of AAV-based *PIGA*-targeting vectors used for assays shown in Figures 5B and 6A. *PIGA* 5′ and *PIGA* 3′ represent homology arms designed for the targeting of human *PIGA* gene. The diagram is not drawn to scale. (**B**) Numbers of G418-resistant colonies obtained by the infection of AAV vectors. The cell lines indicated in the graphs were infected with the AAV-based *PIGA*-targeting vectors depicted in (A) and selected with G418 until visible colonies were formed. Colonies were stained with crystal violet and then counted (mean ± s.e.m.; n = 3). For abbreviations, refer to legend for Figure 1. *Significantly higher than *hEF-1α*, CAG, SV40, HSV-TK, *hACTB*-R, and N.P. (*p* = 0.01 versus *hEF-1α*; *p*<0.001 versus the others). **Significantly higher than any of the other promoters and controls (*p*<0.001 for all pairs). ***Significantly higher than *hACTB*, *hEF-1α*, CAG, HSV-TK, *hACTB*-R, and N.P. (*p* = 0.003 versus *hACTB*; *p* = 0.02 versus *hEF-1α*; *p*<0.001 versus the others). *p*-values were determined based on Scheffe’s post hoc test.

AAV vectors have been frequently used as a platform to generate gene targeting vectors that permit gene editing in human cell lines. In the process of AAV-mediated gene targeting in the majority of human cell lines, a large number of antibiotic-resistant colonies must be recovered following introduction of targeting vectors into cells and screened for gene-targeted cell clones. The use of a highly active promoter to drive an antibiotic resistance gene within AAV-based targeting vectors may be advantageous to secure a large number of colonies upon antibiotic selection. To address which promoter induces efficient formation of antibiotic-resistant colonies, we constructed six different AAV-based targeting vectors against the human *PIGA* gene, differing only in the promoters regulating the Neo^R^ gene (Fig. 5A). The *PIGA* gene, known to be indispensable for glycosylphosphatidyl inositol (GPI) anchor biosynthesis [43], [44], resides on the X chromosome. Thus, targeted disruption of a *PIGA* allele in diploid male cells is readily detectable by negative staining of GPI-anchored proteins or GPI anchor itself on the cell membrane [45], [46]. In this colony formation assay, we thus employed three near-diploid cell lines of male origin, HCT116, DLD-1, and HT-1080, to enable subsequent evaluation of gene targeting efficiencies achieved with these *PIGA*-targeting vectors in the same cell lines.

The three cell lines were infected with aforementioned AAV-based *PIGA*-targeting vectors and propagated in the presence of G418. The use of the CMV promoter to drive the Neo^R^ gene resulted in significantly more efficient G418-resistant colony formation than the other five promoters in all three cell lines, except that the differences between the CMV and the *hACTB* promoters in the HCT116 cell line and the CMV and the SV40 promoters in the HT-1080 cell line did not reach statistical significance (Fig. 5B). In all three cell lines, negative control vectors (*hACTB*-R and N.P.) yielded small numbers of colonies after G418 selection. However, in another control experiment, these cell lines were infected with an AAV vector without the Neo^R^ gene, and G418 selection of the infected cells resulted in no colony formation (data not shown). In addition, a dose response analysis of these cell lines with various amount of G418 (Fig. S4) suggested that the antibiotic selections in our colony formation assay were carried out with sufficient G418 concentrations, but a trace amount of Neo^R^ produced by the control vectors resulted in slight colony formation under G418 selection. Collectively, this colony formation assay demonstrated that the CMV promoter elicits a large number of G418-resistant colonies in comparison with the other constitutive promoters, although we again observed substantial variation depending on recipient cell lines in the efficiency of G418-resistant colony formation with each promoter.

### Distinct gene targeting efficiencies achieved by a series of AAV-based targeting vectors in which different promoters regulate Neo^R^

**Figure 6:**
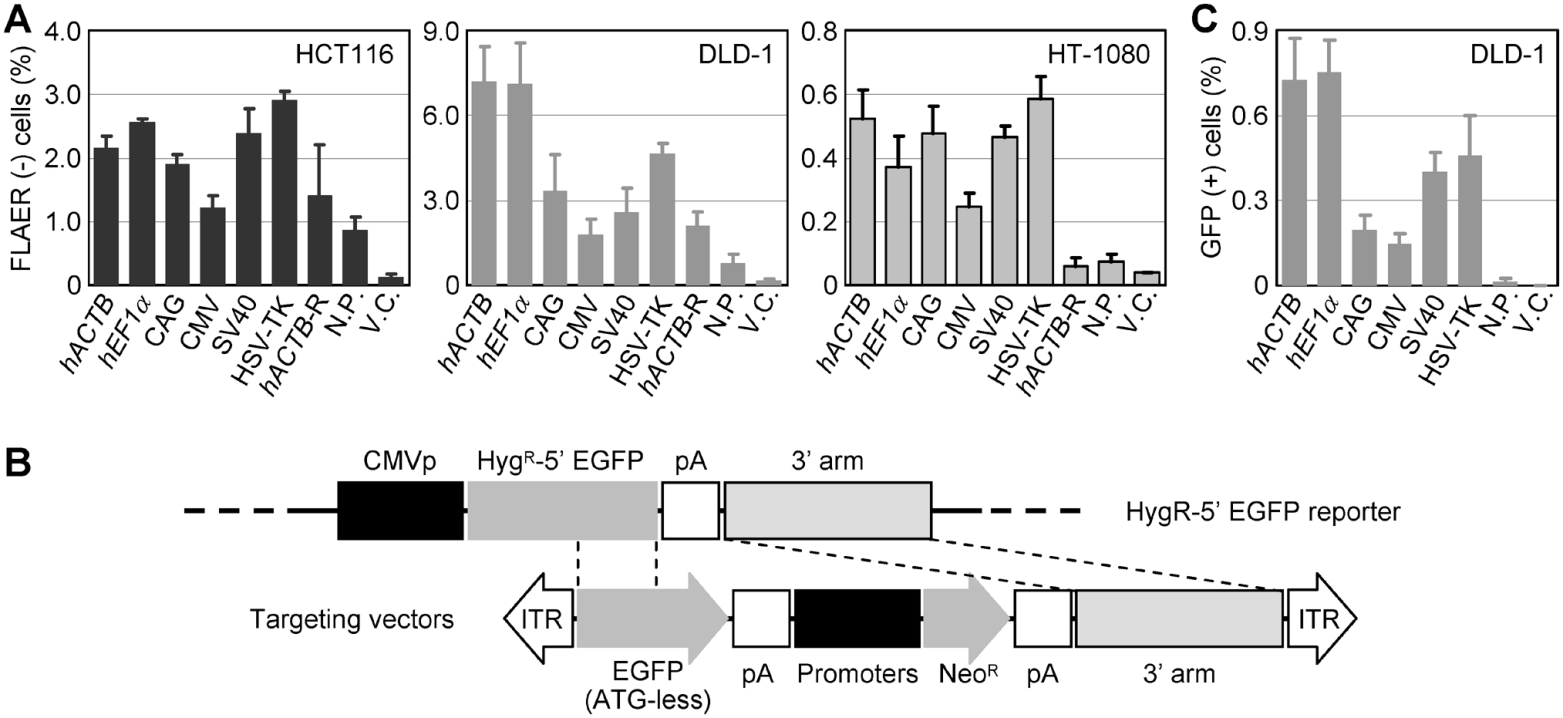
The impact of a promoter driving Neo^R^ expression within AAV-based targeting vectors on gene targeting efficiency. (**A**) *PIGA* gene targeting efficiency achieved with AAV-based targeting vectors carrying various promoters. The cell lines denoted in the graphs were infected with the targeting vectors shown in Figure 5A, selected with G418, processed for fluorescence-labeling of GPI-anchors with FLAER, and analyzed by fluorescence FCM. The ratios of FLAER-negative cells which represent *PIGA* gene-targeted cells are shown (mean ± s.e.m.; n = 3). (**B**) Schematic representation of an experimental system determining gene targeting efficiency using a Hyg^R^–5′ EGFP fusion reporter gene. The Hyg^R^–5′ EGFP reporter vector (top) was introduced into DLD-1, and a cell clone stably expressing the Hyg^R^–5′ EGFP gene was established. This reporter clone was then infected with AAV-based targeting vectors harboring various promoters (bottom), selected with G418, and FCM-analyzed. The diagram is not drawn to scale. (**C**) The gene targeting efficiencies determined based on the Hyg^R^–EGFP reporter system. Shown are the ratios of GFP positive cells which represent the frequency of homologous recombination events occurring between the reporter and the targeting vectors (mean ± s.e.m.; n = 3). An unrelated AAV vector harboring the Neo^R^ gene was used for V.C. For abbreviations, refer to legend for Figure 1.

It is not well known whether the use of distinct constitutive promoters to drive an antibiotic resistance gene within an AAV-based targeting vector results in different gene targeting efficiencies (the ratio of homologous to random integration of a targeting vector into the genome). To address this issue, we infected the HCT116, DLD-1, and HT-1080 cell lines with the aforementioned AAV-based *PIGA*-targeting vectors carrying various promoters directing Neo^R^ (Fig. 5A), and the *PIGA* gene targeting efficiency was quantified as described previously [24]. This assay revealed that respective promoters driving the antibiotic resistance gene within the targeting vector achieve distinct gene targeting efficiencies, and the efficiencies also vary depending on recipient cell lines (Figs. 6A and S5A). However, in all three cell lines, the use of the CMV promoter resulted in the lowest *PIGA* gene targeting efficiency among the six constitutive promoters tested in this study. The *PIGA* gene targeting efficiency in HCT116 and DLD-1 achieved with the CMV promoter in this study appeared to be somewhat lower than those obtained in our previous study [24], possibly because of different lots of reagents including fetal bovine serum as well as different settings of FCM parameters utilized in these studies. Interestingly, infection of HCT116 and DLD-1 with the negative control vectors (*hACTB*-R and N.P.) elicited *PIGA* gene targeting with decent efficiencies comparable with those achieved by the use of the CMV promoter, despite that the CMV promoter and the negative controls induced remarkably different levels of long-term gene expression and G418-resistant colony formation (Figs. 2 and 5). This result suggests that the transcriptional activity of the promoter is not the only major determinant of gene targeting efficiencies achieved by these AAV-based targeting vectors.

To further characterize the relationship between gene targeting efficiency and the promoter regulating an antibiotic resistance gene in an AAV-based targeting vector, we employed another molecular system bearing a fused Hyg^R^–5′ EGFP reporter gene to quantify gene targeting efficiency [25]. We constructed targeting vectors in which one of the six constitutive promoters regulates the Neo^R^ gene, and used the resultant targeting vectors to reconstitute a functional EGFP coding sequence via homologous recombination with a Hyg^R^–5′ EGFP reporter vector which had been transduced into the DLD-1 cell line (Fig. 6B). FCM-based detection of GFP positive cells indicated that the CMV promoter elicits relatively low percentage of homologous recombination events in this reporter system compared with the other constitutive promoters (Figs. 6C and S5B). Overall, our current study suggested that the CMV promoter, which provides strong and prolonged transcription from AAV vectors, elicits relatively low frequency of gene targeting when used in AAV-based targeting vectors to drive the Neo^R^ gene.

## Discussion

In the present study, we demonstrated that transcriptional activities conferred by six constitutive promoters within AAV vectors vary depending on recipient cell lines. However, the CMV promoter within an AAV vector directed stronger transient and long-term gene expression compared with the other five constitutive promoters in three out of four cell lines. In a previous study, we also demonstrated that the CMV promoter in plasmid vectors directs a prominently high level of transient gene expression, but such gene expression does not persist for a long period of time in human cell lines [4]. The finding that the CMV promoter directs more stable gene expression in AAV vectors than in plasmids is consistent with a commonly accepted notion that AAV vectors allow sustained expression of exogenous genes within cells [18], [19].

As compared to three other cell lines (HCT116, DLD-1, and HT-1080), the MCF-10A cell line exhibited a fairly different pattern of gene expression driven by the six constitutive promoters; the CMV promoter directed relatively strong GFP expression in a transient state, but this GFP expression reduced to a level among the weakest in the six constitutive promoters in four weeks. It is unclear why MCF-10A is apparently different from the other three cell lines in regard to the levels of gene expression driven by individual promoters. One of the potential explanations for this is the tissue tropism of AAV serotype 2 from which our AAV vectors were derived [47], [48]. In addition, even cell lines originating from the same tissue type may exhibit distinct patterns of gene expression obtained with respective promoters, because a previous study demonstrated that the *hEF-1α* promoter induce a higher long-term gene expression than the CMV promoter in a colon cancer cell line HT-29 [21], which is contrary to our data with the HCT116 and the DLD-1 colon cancer cell lines. Furthermore, it is also speculated that oncogenic properties of recipient cells might affect transcriptional activities of the promoters, as MCF-10A is the only noncancerous cell line in the four cell lines analyzed in this study. However, gene expression driven by respective promoters in these cell lines may vary depending on the serotype of AAV vectors employed, in view of previous studies indicating distinct gene transduction abilities and tropisms of AAV vectors based on different serotypes [49], [50].

Consistent with the ability of the CMV promoter to introduce strong and prolonged gene expression in the current experimental setting, the CMV promoter, when placed upstream of the Neo^R^ gene in an AAV vector, permitted the formation of the largest number of G418-resistant colonies from the HCT116, DLD-1, and HT-1080 cell lines among the tested promoters. Meanwhile, an AAV-based targeting vector with the CMV promoter regulating the Neo^R^ gene yielded the lowest *PIGA* gene targeting efficiency. Gene targeting efficiency is determined by the number of gene-targeted clones divided by the number of clones transduced with the targeting vector (*i.e.*, antibiotic-resistant clones) upon introduction of the targeting vector into cells. Thus, our experimental data indicate that the use of the CMV promoter to express Neo^R^ did not increase *PIGA* gene-targeted clones to the extent that it increased G418-resistant clones. A possible explanation for this is that the endogenous transcriptional machinery of the *PIGA* gene serves to drive the Neo^R^ gene upon homologous integration of the targeting vector into the genome. In this case, *PIGA* gene-targeted clones may emerge even without a functional promoter driving Neo^R^ expression within the vector. Indeed, we observed substantial efficiency of *PIGA* gene targeting upon infection of cells with *PIGA*-targeting vectors carrying no functional promoters (*hACTB*-R and N.P.). Furthermore, when we utilized a gene targeting model in which the transcriptional machinery from the target gene was blocked upstream of the Neo^R^ gene (Fig. 6B), the use of N.P. control as a promoter within the targeting vector resulted in a minimal frequency of gene targeting (Fig. 6C). These results provide evidence that the expression levels of target genes may have an impact on gene targeting efficiency achieved by AAV-based targeting vectors in a standard gene targeting design (*i.e.*, without an additional polyadenylation site upstream of the Neo^R^ gene). In addition, our data also suggest that the use of the CMV promoter to drive an antibiotic resistance gene in AAV-based targeting vectors results in low gene targeting efficiency as a trade-off with a high yield of antibiotic-resistant colonies. Thus, in order to achieve efficient gene targeting, the use of a strong constitutive promoter such as the CMV promoter may not always be preferable to drive an antibiotic resistance gene in AAV-based targeting vectors.

Although the use of the CMV promoter led to the formation of the largest number of G418-resistant colonies and the lowest gene targeting efficiency in our experimental system, there was no clear inverse correlation between *PIGA* gene targeting efficiencies and the numbers of G418-resistant colonies obtained by the use of the six constitutive promoters (r = −0.7944, not significant in HCT116; r = −0.4948, not significant in DLD-1; and r = −0.8330, *p* = 0.04 in HT-1080). Similarly, there was no clear inverse correlation between gene targeting efficiencies and long-term GFP signal intensities induced by respective promoters in AAV-based vectors (r = −0.8211, *p*<0.05 in HCT116; r = −0.3131, not significant in DLD-1; and r = −0.4443, not significant in HT-1080). These data suggest a complicated mechanism determining gene targeting efficiency achieved by AAV-based targeting vectors; gene targeting efficiency may be affected by unknown, additional factors besides the activities of the promoters regulating an antibiotic resistance gene in targeting vectors and the expression levels of target genes.

Although gene targeting in human cell lines is useful in enabling functional analyses of human genes in a clean genetic background, this technology remains difficult to achieve, primarily because the majority of human cell lines have low gene targeting efficiencies with the exception of a few model cell lines [51], [52]. For gene targeting in human cell lines, AAV-based targeting vectors are frequently used as they are known to induce higher frequencies of gene targeting compared with plasmid-based targeting vectors [22]. Another advantage of AAV-based targeting vectors is that they do not actively introduce double strand breaks into the genome, thus likely accumulating a low frequency of nonspecific genetic alterations in the genome during the process of gene targeting. Given its favorable properties, AAV-mediated gene targeting has been used in many studies conducting gene editing in human cell lines [23], [53]–[61]. Nonetheless, further improvements in gene targeting efficiency are needed to develop a simple procedure of AAV-mediated gene targeting readily applicable to a broad range of human cell lines.

The use of a promoter-trap system is another strategy to improve the efficiency of gene targeting by enriching gene-targeted clones [62]–[64]. In this system, no promoter is placed at the upstream of an antibiotic resistance gene within a targeting vector, and the endogenous promoter of a target gene is trapped and exploited to confer antibiotic resistance. However, promoter-trap strategy may not be readily applicable to some types of gene targeting, *e.g.*, targeting of a gene regulated by a low-active promoter not potent enough to confer antibiotic resistance, and the editing of the enhancer/promoter region of a gene. In addition, promoter-trap strategy may not be preferred in the targeting of a gene whose expression is suppressed depending on cellular contexts or environments. In such cases, targeting vectors carrying a constitutive promoter to drive an antibiotic resistance gene are used, and the promoter should be carefully selected in order to achieve efficient gene targeting in human cell lines. Our current study provides an initial clue to the identification of promoters optimal for use in AAV-based targeting vectors.

## Supporting Information

Figure S1 **Construction of AAV vectors carrying the EGFP gene downstream of various constitutive promoters.** In diagrams, “Promoters” indicates the position where one of the six constitutive promoters was incorporated. “SspI” and “NotI” indicate positions cleaved by respective restriction enzymes. Kan^R^/Neo^R^, neomycin phosphotransferase gene.
(PDF)

Figure S2 **Representative dot plots showing transient GFP expression in AAV infectants.** Cell lines indicated at the top were infected with AAV vectors carrying the EGFP gene regulated by the promoters shown to the left, and FCM-analyzed after 2-days culture. FL1-A on X-axes and FL2-A on Y-axes represent the intensities of GFP and autofluorescence signals, respectively. Percentage of GFP positive cells in each infectant is denoted in dot plot.
(PDF)

Figure S3 **Representative dot plots showing correlation of the MOI of an EGFP-expressing AAV vector with GFP expression in infected cells.** Cell lines indicated at the top were infected with the AAV vector at the MOIs shown to the left, and processed for fluorescence FCM analyses two day later. Data were acquired and presented in the same fashion with Figure S2.
(PDF)

Figure S4 **G418 dose response curves of the cell lines analyzed in the colony formation assay.** Each parental cell line was plated in 75-cm^2^ flasks at densities of 2,000 cells/flask (HCT116 and DLD-1) or 500 cells/flask (HT-1080), and selection with G418 at indicated concentrations was started immediately. A few weeks later, visible colonies in each flask were fixed, stained, and counted (mean ± s.e.m.; n = 3).
(PDF)

Figure S5 **Representative results of two distinctive FCM-based assays for the quantification of gene targeting efficiencies.** Each dot plot indicates the efficiency of *PIGA* gene targeting (A) or that of homologous recombination within the Hyg^R^–EGFP constructs (B) elicited by the use of each constitutive promoter. Denoted at the top are cell lines used for the assays. Listed to the left are promoters placed in an AAV-based targeting vector to drive the Neo^R^ gene. Percentages of FLAER-negative (A) or GFP positive (B) cells are noted in dot plots.
(PDF)

Table S1 **Oligonucleotide Primers used for qRT-PCR.**
(PDF)
