# Supplementary figures and images for: A Comparative Analysis of Constitutive Promoters Located in Adeno-Associated Viral Vectors
Source: PLoS One. 2014 Aug 29;9(8):e106472. doi: 10.1371/journal.pone.0106472 (PMC4149579; doi:10.1371/journal.pone.0106472)

**Figure S1**

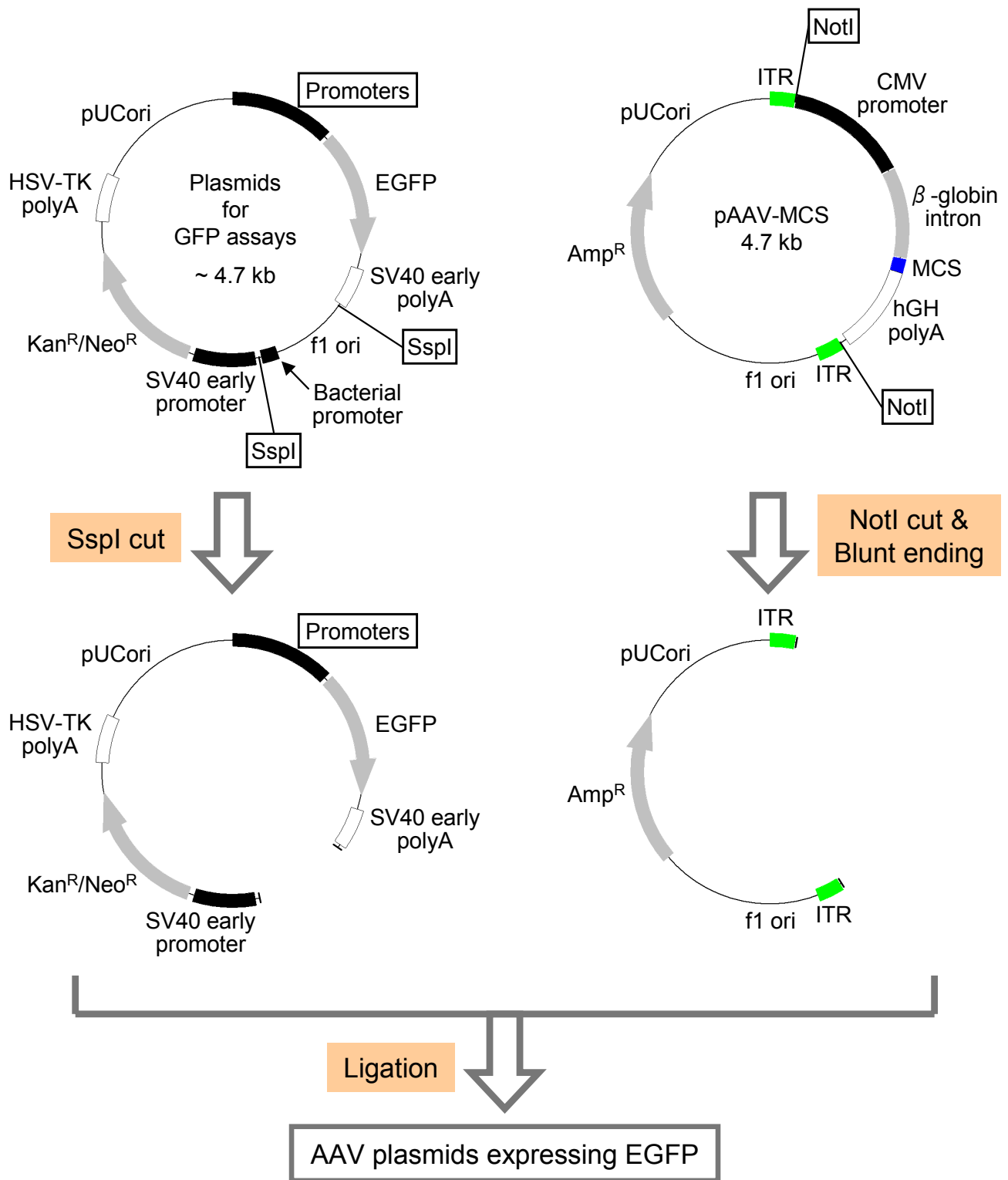

Supplement: Figure S1 — Construction of AAV vectors carrying the EGFP gene downstream of various constitutive promoters. In diagrams, “Promoters” indicates the position where one of the six constitutive promoters was incorporated. “SspI” and “NotI” indicate positions cleaved by respective restriction enzymes. KanR/NeoR, neomycin phosphotransferase gene. (PDF) [file pone.0106472.s001.pdf]

**Figure S2**

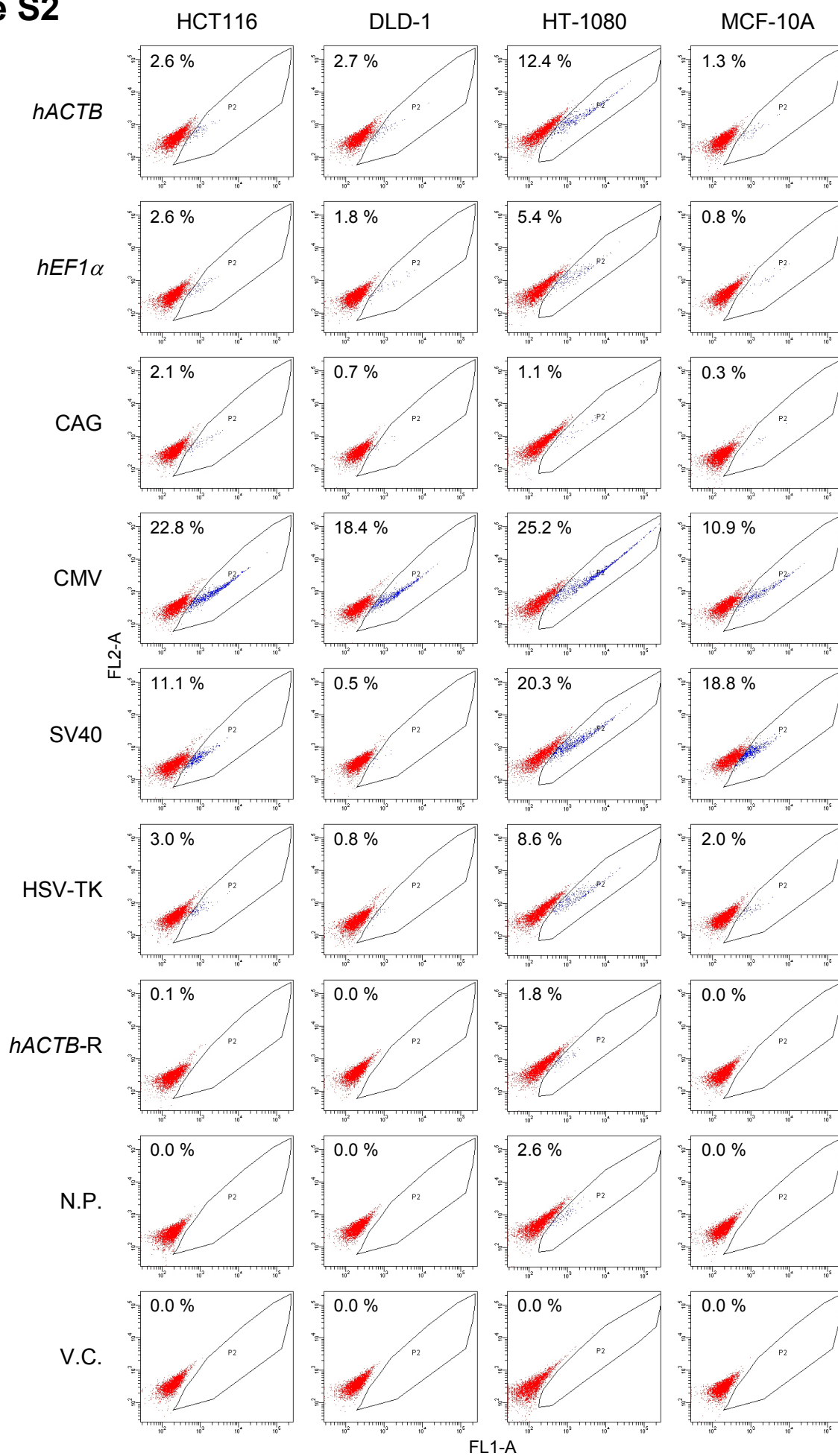

Supplement: Figure S2 — Representative dot plots showing transient GFP expression in AAV infectants. Cell lines indicated at the top were infected with AAV vectors carrying the EGFP gene regulated by the promoters shown to the left, and FCM-analyzed after 2-days culture. FL1-A on X-axes and FL2-A on Y-axes represent the intensities of GFP and autofluorescence signals, respectively. Percentage of GFP positive cells in each infectant is denoted in dot plot. (PDF) [file pone.0106472.s002.pdf]

**Figure S3**

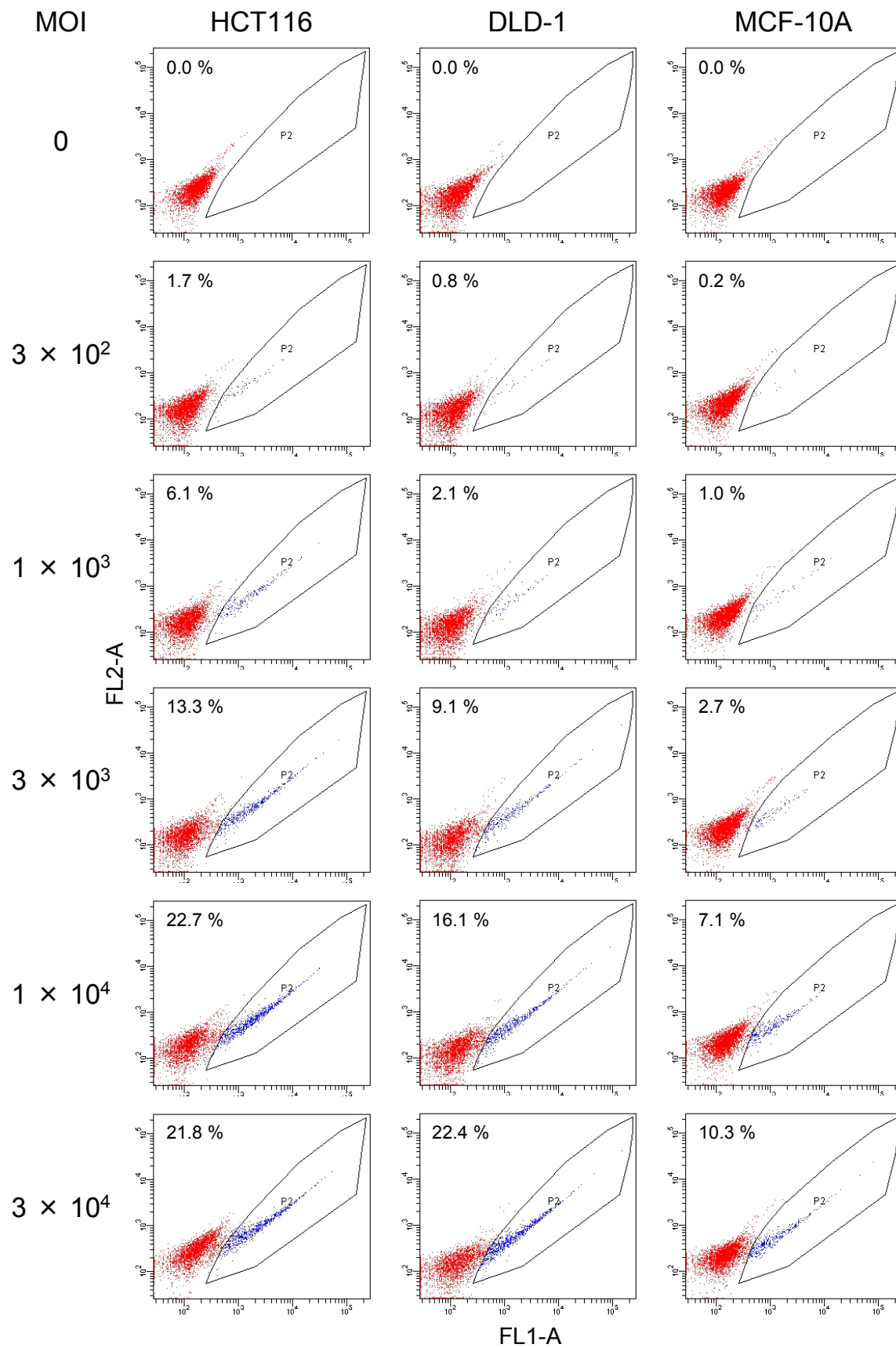

Supplement: Figure S3 — Representative dot plots showing correlation of the MOI of an EGFP-expressing AAV vector with GFP expression in infected cells. Cell lines indicated at the top were infected with the AAV vector at the MOIs shown to the left, and processed for fluorescence FCM analyses two day later. Data were acquired and presented in the same fashion with Figure S2. (PDF) [file pone.0106472.s003.pdf]

**Figure S4**

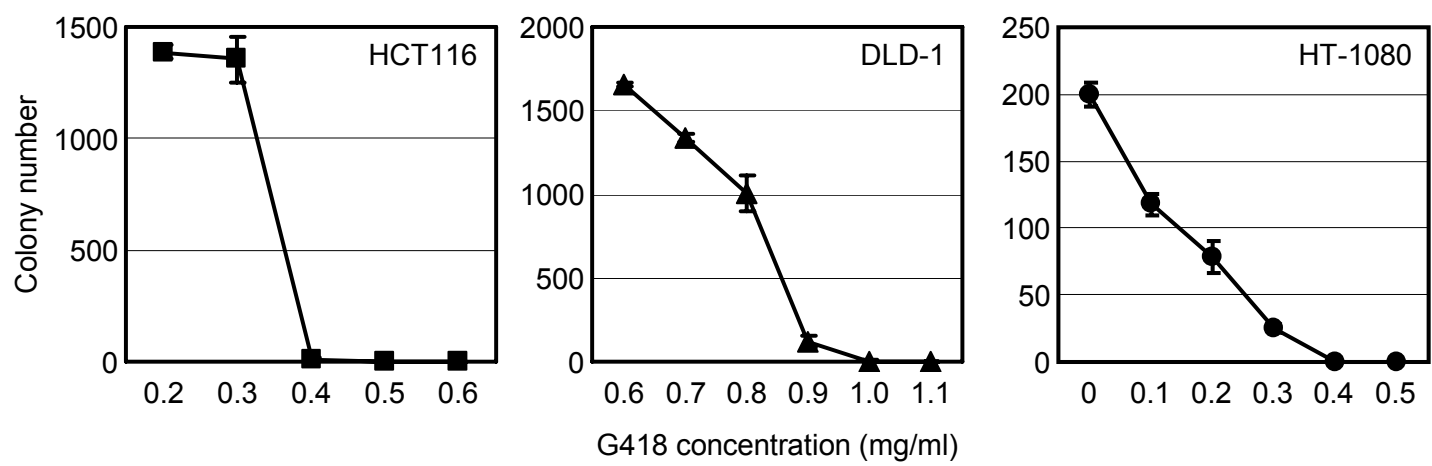

Supplement: Figure S4 — G418 dose response curves of the cell lines analyzed in the colony formation assay. Each parental cell line was plated in 75-cm2 flasks at densities of 2,000 cells/flask (HCT116 and DLD-1) or 500 cells/flask (HT-1080), and selection with G418 at indicated concentrations was started immediately. A few weeks later, visible colonies in each flask were fixed, stained, and counted (mean ± s.e.m.; n = 3). (PDF) [file pone.0106472.s004.pdf]

**Figure S5**

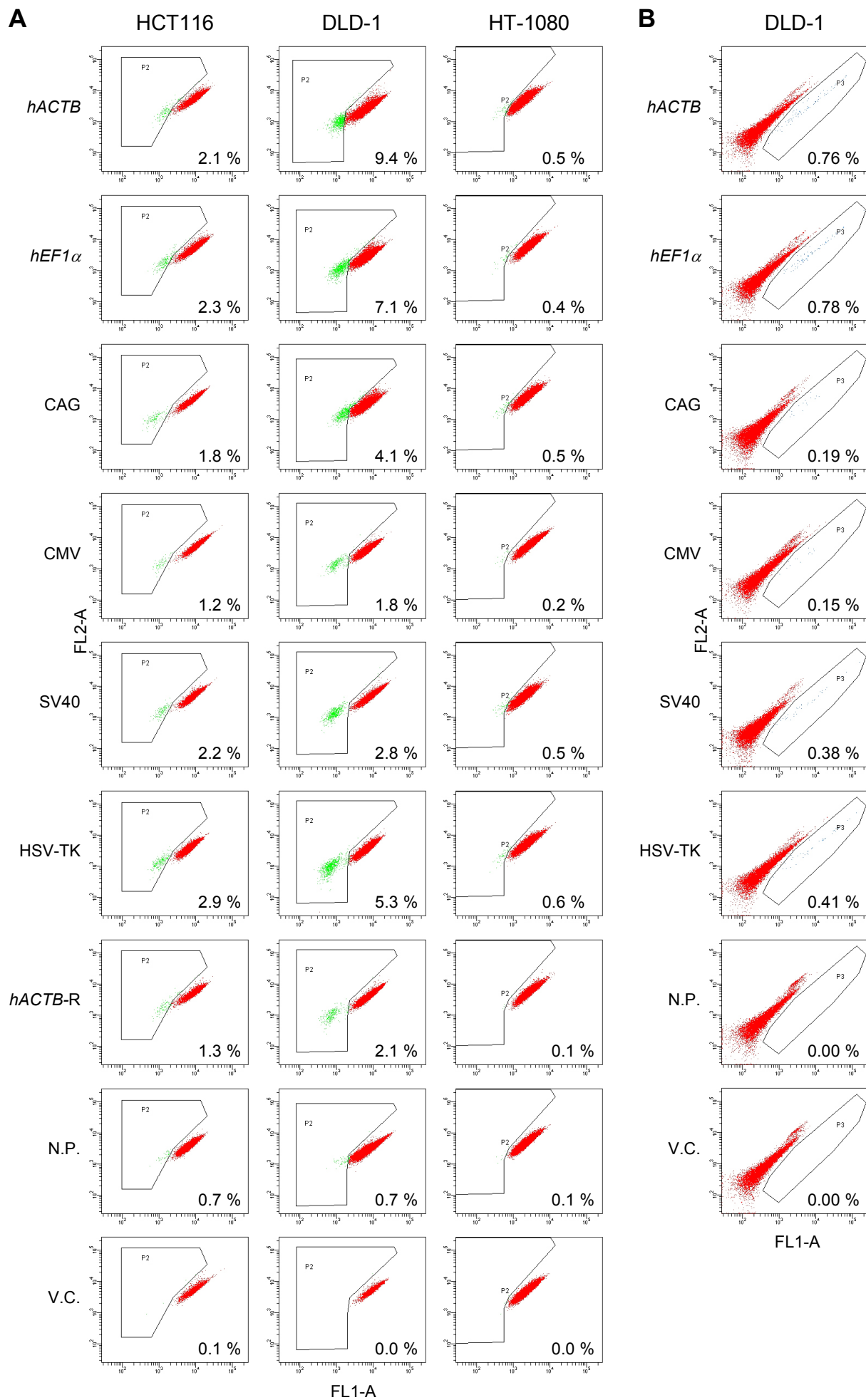

Supplement: Figure S5 — Representative results of two distinctive FCM-based assays for the quantification of gene targeting efficiencies. Each dot plot indicates the efficiency of PIGA gene targeting (A) or that of homologous recombination within the HygR–EGFP constructs (B) elicited by the use of each constitutive promoter. Denoted at the top are cell lines used for the assays. Listed to the left are promoters placed in an AAV-based targeting vector to drive the NeoR gene. Percentages of FLAER-negative (A) or GFP positive (B) cells are noted in dot plots. (PDF) [file pone.0106472.s005.pdf]
