# Supplementary material for: A Comparative Analysis of Constitutive Promoters Located in Adeno-Associated Viral Vectors
Source: PLoS One. 2014 Aug 29;9(8):e106472. doi: 10.1371/journal.pone.0106472 (PMC4149579; doi:10.1371/journal.pone.0106472)
Supplement: Table S1 — Oligonucleotide Primers used for qRT-PCR. (PDF) [file pone.0106472.s006.pdf]

Table S1. Oligonucleotide Primers used for qRT-PCR

Primers	*Sequences*
hCDKN2A fwd	AACGCACCGAATAGTTACGG
hCDKN2A rev	ACCAGCGTGTCCAGGAAGCC
hGAPDH fwd	GAGTCAACGGATTTGGTCGT
hGAPDH rev	GACAAGCTTCCCGTTCTCAG
